# Imprecise descriptions of *Passiflorariparia* Martius ex Masters led to redundant descriptions as *P.emiliae* Sacco, *P.crenata* Feuillet & Cremers, *P.pergrandis* Holm-Nielsen & Lawesson and *P.fernandezii* Escobar

**DOI:** 10.3897/phytokeys.117.30672

**Published:** 2019-02-04

**Authors:** Maxime Rome, Geo Coppens d’Eeckenbrugge

**Affiliations:** 1 Univ. Grenoble Alpes, CNRS, SAJF, F-38000 Grenoble, France; 2 CIRAD, UMR AGAP, Avenue Agropolis, 34398 Montpellier, France; 3 AGAP, Univ Montpellier, CIRAD, INRA, Montpellier SupAgro, Montpellier, France

**Keywords:** Passifloraceae, subgenus *Passiflora*, series *Laurifoliae*

## Abstract

*Passiflorariparia* was incompletely described by Masters, who cited specimens of Martius and Spruce. While *Spruce 2191*, the unique syntype with an observable corona, exhibits a reduced outermost series of filaments, the accompanying iconography represents two equal outer series. Later descriptions have neither added significant information nor corrected the inconsistency in the corona description, so that four closely related species have been distinguished on the basis of traits not properly documented for *P.riparia*: *P.emiliae* (unequal outer series of filaments), *P.crenata* (bract color), *P.pergrandis* (flower size and sepal awn length), and *P.fernandezii* (hypanthium pubescence and shape). The present study compares (i) the descriptions of the above-mentioned taxa and (ii) 43 associated vouchers, as well as live specimens from two associated *P.crenata* populations. These and other specimens were georeferenced for a comparison of their distribution and habitat. Of the five *P.riparia* descriptions found in floras, only that of the Flora of Ecuador appears clearly divergent, corresponding in fact to *P.tolimana*. Those of the four other taxa only differ by unequal corona filaments (except for *P.crenata*) and the pubescence of floral parts. However, 22 vouchers associated with all these descriptions (including 16 for *P.riparia*), as well as the live specimens, share both these traits; the other 21 vouchers were uninformative and/or could not be assigned to any of the five species. The wider sample of 62 specimens indicates no significant differences in either geographic or in climatic distribution (lowlands of the Amazon basin), and a marked preference for riparian habitats. Thus, their very close morphology and ecology justify the placement of *P.emiliae*, *P.crenata*, *P.pergrandis* and *P.fernandezii* as synonyms of *P.riparia*, designating *Spruce 2191* as epitype. The most similar species, *P.ambigua* (20 specimens mapped), differs in corolla and bract color, as well as a distribution centered along the tropical Andes of South America and in Central America, in more diverse habitats.

## Introduction

Five subgenera are currently recognized in the genus *Passiflora* L.: *Passiflora, Astrophea* (DC.) Mast., *Decaloba* (DC.) Rchb., *Deidamioides* (Harms) Killip and *Tetrapathea* (DC.) P. S. Green. SubgenusPassiflora, rich with more than 250 species, is characterized by large flowers with a corona made of several rows of filaments. Its supersection Laurifoliae (Cervi) Feuillet & J. M. MacDougal includes several series, organized around a clear morphological pattern (Rome and Coppens d’Eeckenbrugge 2017). In particular, series *Laurifoliae*Killip (1938) ex Cervi (1997) forms a very homogenous morphological group, with a very difficult taxonomy (Killip 1938, Holm-Nielsen et al. 1988). With the recently described *P.gustaviana* Ocampo & Molinari (Ocampo and Molinari 2017), it is composed of 25 species including glabrous to pubescent plants, whose stems are terete to angular and sometimes corky on old parts; leaves are unlobed, oblong to oblong-lanceolate, not peltate, with entire to glandular-serrulate margins, biglandular petioles; and stipules are setaceous or linear, and early deciduous. Their three bracts, free at base, with entire or serrulate-glandular margins, are more than 1 cm long. Their flowers are pendent, usually large and showy, often fragrant, with a short hypanthium and two campanulate series of long external filaments, and a variable number of series of reduced internal filaments (Rome and Coppens d’Eeckenbrugge 2017).

According to Killip (1938), *Passiflorariparia* Martius ex Masters appears to be the commonest representative of the series *Laurifoliae* in the upper and middle Amazon basin. Its original description by Masters (1872), in Flora Brasiliensis, refers to two syntypes: *Martius 3228*, from the mouth of the Madeira River in the Upper Amazonas, and *Spruce 2191* (the latter cited by Masters with an exclamation mark), collected from São Gabriel da Cachoeira, near the Rio Negro. The number of series of external corona filaments is not mentioned in Masters’ description. In the accompanying botanical iconography, flowers appear to have one series of long filaments, whereas a schematic longitudinal section shows two equal series of external filaments. This detail has become problematic since the relative length of the two external filament series became an important diagnostic trait when Killip (1938) created the series.

Martius’ specimen consists of two samples with much deteriorated flowers, which do not allow assessing the length of filament series. However, one of them holds a brief note in Latin handwritten by Martius, with incomplete information about the filaments: “Corona exterior serie simplici constat filorum alborum quae vittis quadratis violaceis picta sunt atque compressa; interior similis crassior”. This indicates the existence of two outer series, the inner one with stronger filaments, but gives no direct information on their relative length. The flower of *Spruce 2191*, the specimen examined by Masters, has two very unequal series of external filaments, the outermost being reduced, with few short filaments. Thus, the illustration accompanying Masters’ description of *Passiflorariparia* reflects misinterpretation of the syntypes concerning an important diagnostic trait in the series. However, this anomaly was not corrected in the successive re-descriptions of the species (Killip 1938, Tillett 2003, Cervi 1997). The resulting confusion is responsible for the creation of several species that are closely similar or identical to the syntypes of *Passiflorariparia*.

Thus, in 1966, Sacco described *Passifloraemiliae* Sacco in the Boletim do Nacional Museum (Rio de Janeiro), from two Kuhlmann specimens that had been first determined as *P.riparia*. Indeed, as in *Spruce 2191*, the Kuhlmann specimens correspond to Masters’ drawing of *P.riparia*, with the exception of their two unequal outer series of filaments. This is why Sacco (1966) provided a key where *P.riparia* is differentiated from *P.emiliae* by two subequal outer series of filaments.

*Passifloracrenata*Feuillet and Cremers (1984) was described as a French Guiana endemic, very similar to *P.riparia*. It was differentiated by the white color of its bracts (a trait not precisely described for *P.riparia*) and by its solitary flowers, in contrast to those of *P.riparia*, which are clustered on short lateral stems. The original description of *P.crenata* mentions two outer series of the same length as the sepals, however both the holotype and the illustration provided by Feuillet and Cremers (1984) show two very unequal outer series of filaments, the outermost appearing much shorter and thinner.

In the Flora of Ecuador, Holm-Nielsen et al. (1988: 116) designated *Martius 3228* as the lectotype of *P.riparia* and referred the other syntype, *Spruce 2191*, to *P.ambigua* Hemsl. With two glands placed above the middle of petiole (at the apex in the reference vouchers), their description of *P.riparia* is clearly different from the syntypes and the original description. Moreover, their description indicates a confusion between the innermost series of filaments and the operculum. Indeed, the authors describe “a nectar ring 0.5 cm long, recurved margin minutely sinuate to lobulate” and an “operculum 1 cm, erect, entire, borne just below corona”. In fact, the “nectar ring” corresponds to the operculum as observed in the series *Laurifoliae*; and the part named “operculum” by Holm-Nielsen et al. is the same innermost series of filaments, oriented towards the androgynophore and covering the entrance of the hypanthium, that exists in *P.tolimana* Harms, *P.kapiriensis* Rome and Coppens, and other species of the series (Rome and Coppens d’Eeckenbrugge 2017). In the herbarium specimen *Knapp 6242* cited by Holm-Nielsen et al. (1988), these inner filaments appear more or less fused together, forming an erect tubular structure. On the next page, Holm-Nielsen *et al*. described *P.ambigua*, including *P.emiliae* as a synonym of this species. Furthermore, in their determination key, they distinguished *P.pergrandis*Holm-Nielsen and Lawesson (1987), another species of series *Laurifoliae*, from *P.riparia* by the awn of the sepal not exceeding its apex (vs. an awn exceeding the sepal apex). However, this trait is found in Spruce’s syntype of *P.riparia* (which they assigned to *P.ambigua* instead of *P.riparia*).

In 1989, Escobar described *Passiflorafernandezii* Escobar as a close Bolivian relative of *P.riparia*: “*P.fernandezii* most closely ressembles *P.riparia* […], but differs from it by the pubescence and shape of the hypanthium. Both of these characters are variable in collections ascribed to that species […] so that further study of the *Passiflorariparia* complex is needed.”

Here, we revise the different treatments of *P.riparia* and compare them with the descriptions and types of *P.emiliae*, *P.crenata*, *P.pergrandis*, and *P.fernandezii*, and re-examine the other vouchers associated to them by the different authors of these species. Thus, we can demonstrate that, based on the textual descriptions of *P.riparia* by Martius and Masters and the observation of both syntypes, *Martius 3228* and *Spruce 2191*, as well as the polymorphism observed on the reference vouchers mentioned in the different treatments, there is no ground for differentiating these five species. We also verify that none of these five taxa can be differentiated by its adaptation, using label information and a multivariate analysis of climates in their respective ranges, while observing that *P.ambigua* is marginally sympatric with them, less strongly related to riparian habitats, and adapted to a wider range of climates, particularly in tropical highlands. Consequently, we place *P.emiliae*, *P.crenata*, *P.pergrandis* and *P.fernandezii* in synonymy of *P.riparia*, and provide a more complete and precise description that takes into account the pan-Amazonian geographical distribution of this species and its attendant polymorphism.

## Materials and methods

A first comparison confronts the descriptions of *P.riparia* by Masters (1872), Killip (1938), Cervi (1997), Tillett (2003), Holm-Nielsen et al. (1988), the original description of *P.emiliae* by Sacco (1966), that of *P.crenata* by Feuillet and Cremers (1984), that of *P.pergrandis* by Holm-Nielsen and Lawesson (1987), that of *P.fernandezii* by Escobar (1989). This comparison is focused on traits relative to stems, stipules, petiole, leaves, inflorescences, bracts, hypanthium, sepals, petals, corona (outer and inner series of filaments), operculum, androgynophore, ovary, and fruit. It has been extended to the description of the very similar *P.ambigua* by Hemsley (1902).

A second comparison bears on all available vouchers (43 specimens; see Table 2) associated with the descriptions of any of the first five species by these same authors, focusing on the following traits: petiolar gland position, presence of pseudoracemes, plant pubescence, bract size, color of bracts and perianth, when discernible or mentioned on the voucher label. We also include our own field observations of *P.crenata* in French Guiana, on living material collected from localities indicated in the original description.

Other 94 herbarium specimens have been observed and determined, for a comparative study of the habitat of the same taxa. Some sheets were studied from scans provided by the host herbaria. The complete list, comprising 137 materials initially classified under *P.riparia* or one of its four presumed synonyms or under *P.ambigua*, is listed in the appendix. For the distribution study, only fertile samples allowing unambiguous determination were retained, as well as other good quality reports (including photographs); the *P.crenata* sample from French Guiana was reduced to 11 specimens, to avoid an excessive weight for this taxon. When label information allowed, geographic coordinates were assigned to the voucher using Google Earth and gazetteers, the collecting sites were mapped and a distribution model was developed, using the MAXENT software and 19 bioclimatic variable layers from the Worldclim database at a 2'30" grid resolution (corresponding roughly to 4.4 × 4.6 km at Equator; http://www.worldclim.org/current) (Hijmans et al. 2005). MAXENT identifies potential distribution areas based on their similarity in climatic conditions compared to those at the sites where the species has already been observed, hence modeling where conditions are suitable for their development. It infers the probability distribution of maximum entropy (i.e., closest to uniform) subject to the constraint that the expected value of each environmental variable (or its transform and/or interactions) under this estimated distribution matches its empirical average (Phillips et al. 2006). A logistic threshold value equivalent to the 10^th^ percentile training presence was retained to separate climatically favorable areas from marginally fit areas. Thresholds of 33 and 67% training presence were used to discriminate “very good” and “excellent” climates.

Furthermore, 19 bioclimatic variables corresponding to the collection sites were extracted from the Worldclim database (Hijmans et al. 2005), and those variables that most contributed to the model were submitted to a Principal Component Analysis (PCA) for a comparative analysis of climatic adaptation.

## Results

### Comparative analysis of descriptions

Table 1 compares the descriptions of *Passiflorariparia* with those of its presumed synonyms. The cells highlighted in bold font correspond to the characters that differ from the descriptions of *P.riparia* by Masters (1872) and Killip (1938).

**Table 1.** Comparison of descriptions of *P.riparia*, *P.emiliae*, *P.crenata*, *P.fernandezii*, *P.pergrandis* and *P.ambigua*, with additional information from related iconography (*). Traits diverging from the original description of Masters are highlighted in bold font.

		*P.riparia* (Master, 1872)	*P.riparia* (Killip, 1938)	*P.riparia* (Cervi, 1997)	*P.riparia* (Tillett, 2003)	*P.riparia* (Holm-Nielsen et al., 1988)	*P.emiliae* (Sacco, 1966)	*P.crenata* (Feuillet & Cremers, 1984)	*P.fernandezii* (Escobar, 1989)	P.pergrandis (Holm-Nielsen & Lawesson, 1987)	P.ambigua (Hemsley, 1922)
Stem	shape	terete to striate	terete	terete	–	–	terete, subangular or striate	terete to angular	angular to striate	terete to striate	terete to angular
	pubescence	glabrous	glabrous	glabrous	–	glabrous	glabrous	glabrous	**sparingly pubescent**	glabrous	glabrous
Stipules	shape	setaceous*	linear	linear	linear	setaceous	linear, acute	linear	linear	absent from the type	very slender
	pubescence	glabrous*	glabrous	glabrous	–	glabrous	glabrous	glabrous	–	glabrous	glabrous
	size (cm)	–	0.3 to 0.4 long	0.3 to 0.4 long	0.4 long	0.5 cm	**ca. 1**	**1.5–2.0**	**ca 1 × 0.05**	–	0.5–1.6 × 0.04–0.1
Petiole	length (cm)	1.35–2.25	1.5–2	1.5–2	until 4.5	**3**	1.5–2	1.5–2	1.2–1.8	2–3	2–6
	gland position	below middle	at middle	at middle	at middle	**above middle**	below middle	proximal half	at middle	below middle	at middle
Leaves	base	cordate to rounded	rounded, retuse or narrowed	rounded to retuse	rounded, retuse or narrowed	truncate to obtuse	rounded	rounded, obtuse or slightly cuneate	rounded	obtuse to truncate	rounded or cuneate
	apex	slightly acuminate	abruptly acuminate	abruptly acuminate	abruptly acuminate	acuminate	acuminate, mucronate	acuminate, sometimes mucronate	abruptly acuminate	acuminate	cuspidately acuminate
	margin	entire to serrate	entire to minutely serrulate	entire to minutely serrulate	–	entire	entire	entire	entire	entire	entire to serrulate
	pubescence	glabrous*	glabrous	glabrous	glabrous	glabrous	glabrous	glabrous	**glabrous except for a few scattered trichomes at base on abaxial surface**	glabrous	glabrous
	size (Lxl, cm)	10.8–13.5 × 5.4–8.1	10–15 × 4.5–8	10–15 × 4.5–8	**19 × 9**	**15–17 × 8–9**	10.5–14.5 × 5.5–6.5	6–13 × 2.5–7	**5–9.4 × 2.5–4.2**	**15–20 × 9–10**	**14–23 × 7.2–12**
Inflorescence	type	axillary racemes	on short axillary branches, with no or reduced leaves	–	in axillary branches with or without reduced leaves, occasionally axillary to normal leaves	**solitary**	axillary, solitary or pseudoracemes*	axillary, aggregated at the end of stems	–	a distal bud developing to a short shoot, forming a conspicuous indeterminate inflorescence	solitary, axillary or in pseudoracemes
Bracts	color	colored	reddish	–	reddish	–	–	white	–	–	**green**
	size (Lxl, cm)	large	3–4 × 1.5–2	3–4 × 1.5–2	3–5 × 2–3	4.5 × 1	2.5–3.5 × 1.4–2.2	4–5 × 2–3	1.9–2.4 × 1–1.4	5 × 4	3–6 × 3–4
	pubescence	glabrous*	glabrous	glabrous	–	glabrous	**pubescent**	**pubescent**	**pubescent**	**pubescent**	**pubescent**
Hypanthium	pubescence	pubescent	**glabrous**	**glabrous**	–	**glabrous**	pubescent	–	–	pubescent	pubescent
	shape/size	cylindric campanulate*	cylindric-campanulate	cylindric-campanulate	broadly funnelform	1–1.5 × 2 cm, broadly campanulate	cylindric-campanulate, 1.3 cm long	1 cm long	funnelform, 1.6–2 cm long, 2.4–2.9 cm wide at apex, 1–1.3 cm wide at base	campanulate, 1 × 2 cm	oblate, deeply intruded
Sepals	size (Lxl, cm)	3.75 × 1.8–2.3	4–5 × 2	4–5 × 2	4–5 × 2 cm	4 × 1.5	3.5–4 × 1.3–1.8	**6 × 3**	2.6–3.1 × 1.5–2.1	**6 × 3.5–4**	4–5 × 1.5–1.8
	pubescence	–	glabrous	glabrous	–	glabrous	**pubescent**	**pubescent**	–	**pubescent**	**pubescent**
	color	–	–	–	pinkish white	**lilac**	white	greenish white	–	–	**white outside pink to dark purple inside**
Petals	size (Lxl, cm)	shorter than sepals	4 × 0.8	4 × 0.8	shorter than sepals	**2.5 × 0.5–1**	3–3.5 × 0.8–1	5 × 1–1.5	ca 1.4 × 0.7	5.5–6 × 2	3–4 × 1
	color	–	–	white	pinkish white	**lilac**	white	white	–	white	**white strongly spotted with rose-purple**
Outermost series of filaments	number	2? *	2	2	**2 (rarely 1)**	2	2	2	2	2	2
	shape	thicker than the inner filaments	filamentose, carnose, ca. 2 mm thick	filamentose, carnose, ca. 2 mm thick	the inner to 2 mm thick, filamentous, fleshy, forming a spherical cage around the androgynoecium, outer series more slender	**filiform**	the outermost filiform, the second serie ligulate	large and erected, enlarged at base	filamentous	outer series minutely setaceous, filaments of second series stout, ligulate	the outermost filiform, the second thicker
	color	red striped	white banded with blue or violet	banded with blue or violet and white	banded red to purplish	_	white and red stripped	white and purple stripped	banded with purple	white and dark violet	white banded with red or purple
	relative length	same length*	same length (4–5 cm)	subequal (4–5 cm)	same length (4–5 cm)	**same length (6–7 cm)**	**outer series (1–1.3 cm) shorter than next one (2.5–3.5 cm)**	both as long as sepals	**the outer filaments ca 0.6 cm long, ca 0.4 mm wide, the inner ones 2.3–2.5 cm long, ca 1 mm wide**	**outer series 0.2 cm, second series 5 cm**	**the outermost shorter than the second series (sometimes atrophied)**
Inner series	number	many	many	many	2 or more series within the floral tube	5–6 series	many	4 (more or less distinct)	many	many	many
	length	shorter than outer series, with intermediate series atrophied and the innermost slightly longer	irregular mass of tubercles covering about 6 mm of the height of the tube, the innermost filaments about 2 mm long	irregular mass of tubercles covering about 6 mm of the height of the tube, the innermost series filaments about 2 mm long	2 mm long	**third series 0.5 cm, filiform, then 2 or 3 series 0.2–0.3 cm, filiform, irregularly arranged. The innermost 1 cm, erect, entire, borne just below corona.**	shorter than outer series, with intermediate series atrophied and the innermost slightly longer	short	irregular rows of filaments 0.5–2 mm long in lower half of inner surface	third series close to operculum, minutely tuberculate 1–2 mm	intermediate series atrophied and the innermost slightly longer
Operculum	shape	membranous, horizontal, margin recurved, fimbriate	membranous, horizontal, margin recurved, crenulate	membranous, horizontal, margin recurved, crenulate	horizontal, recurved margin with short, capitellate filaments	0.5 cm, recurved, margin minutely sinuate to lobulate	membranous, horizontal, with margin recurved, crenulate	horizontal	horizontal, membranaceous, nonplicate	menbranaceous, recurved, the margin with short fimbriate filaments	membranous, horizontal, with margin recurved, crenulate
Andro-gynophore	length (cm)	slightly longer than the flower tube.	–	–	–	–	1.5 cm	1 cm	–	–	1.5–2 cm
Ovary	pubescence	yes	yes	yes	yes	yes	yes	yes	yes	yes	yes
Fruit	shape	globose	ovoid to globose	ovoid to globose	ovoid or globose	ovoid	–	ovoid	–	–	ovoid to oblong
	size (Lxl, cm)	larger than a cherry	3–4 × 2.5–3.5	3–4 × 2.5–3.5 cm	**10 × 2.5–5**	**6 × 2.5**	–	**6 × 4**	–	–	**10–12 × 6–7**

As Masters (1872) did not observe living specimens, his original description could not give indications about the size and color of several plant parts. Only the bracts are mentioned as colored, i.e., different from the usual chlorophyll green found in most passionflower species. Masters (1872) specified leaf size, as well as the length of sepals and petals, but gave no direct indication about the length of the outer series of filaments. He ignored Martius’ considerations on the unequal width of the two series. The iconography seems very precise but it is confusing, as it shows a flower section with two equal series of filaments, whereas the other flowers exhibit only one series of long filaments protruding from the corolla.

Based on Peruvian and Brazilian herbarium specimens, the description by Killip (1938) completes that of Masters (1872) with the size of stipules, bracts, inner series of filaments and fruits, and expands the variability of leaves, sepals and petals. It mentions the reddish bract color but not the perianth color. It describes the species with two equal series of filaments.

The description of *P.riparia* by Cervi (1997), based on new specimens from Brazil, Peru and Ecuador is very similar to that of Killip. The only difference is that Cervi (1997) mentions the white color of petals. Another very similar description is that of Tillett (2003) in the Flora of the Venezuelan Guyana, with no mention of examined specimens. It expands further the variability of leaves, perianth color (pinkish white) and fruit size. Although he maintains the presence of two equal series of corona filaments, he notes that the outer one is more slender and that the corona can be reduced to a single series of filaments.

Holm-Nielsen et al. (1988) were the first to mention *P.riparia* in Ecuador, referring to two specimens. Their description differs from the others in many traits: leaf size, the petiole gland position, the type of inflorescence, the color of the corolla, the size of the sepals, the length and shape of the two series of outer corona filaments, and finally the internal structure of the flower with the presence of an internal series of filaments closing the entrance to the nectar chamber. Thus, this description presents many more differences from the original description than those of the five other species examined here: *P.emiliae*, *P.crenata*, *P.pergrandis*, *P.fernandezii*, and even the much more anciently established *P.ambigua*. Its divergence can be visualized by the concentration of bold font in the corresponding column of Table 1. In contrast, the descriptions of *P.emiliae*, *P.crenata*, *P.fernandezii* and *P.pergrandis* are compatible with the earlier descriptions of *P.riparia*, except for pubescence of bracts, hypanthium and sepals, the relative length of the two outermost series of corona filaments, and their wider variation for quantitative traits (dimensions of stipules, leaves, sepals and fruits).

In 1966, Sacco described *P.emiliae* from Brazilian herbarium specimens hitherto classified under *P.riparia*. Holm-Nielsen et al. (1988) considered it as a synonym of *P.ambigua*. A comparison of both species shows that their flower structure is relatively similar, however the bract and perianth colors are clearly different (white bracts and perianth for *P.emiliae* vs. green bracts and dark purple perianth for *P.ambigua*). As compared with *P.riparia* description, that of *P.emiliae* differs by two unequal outer series of filaments (vs. two equal series), as well as pubescent bracts and sepals.

Feuillet and Cremers (1984) described *P.crenata* as an endemic species from French Guiana, distinguishing it from *P.riparia* by its white bracts and the type of inflorescence. Again, the two outer series of filaments are described as equal even though the drawing of the description shows only one outer series of filaments. Bracts and sepals are described as pubescent on their abaxial surface.

The description of *P.fernandezii* (Escobar, 1989), from one herbarium specimen, differs from that of *P.riparia* by two unequal outer series of filaments, a scattered pubescence on stems, leaf abaxial surfaces, and the funnelform hypanthium. It seems to have smaller leaves, bracts and perianth, as compared to the other species in Table 1. However, this difference in leaf size is more likely related to the origin of the specimens used for Escobar’s description as they correspond to the terminal portion of floriferous branches, i. e., pseudoracemes, with shorter nodes and smaller leaves. In fact, the leaves at the base of the samples are about 9–12 cm long, which falls in the range of the other species descriptions.

The description of *P.pergrandis*, only based on the Ecuadorian type specimen, is closely similar to those of *P.crenata* and *P.emiliae*. The qualitative criteria, as leaf shape, the petiole gland position, the pubescence of different parts, the petal color, the hypanthium shape, and the disposition of inner filaments series are identical. The two outer series of filaments are very unequal, the outer elements being 2 mm long (vs. 50 mm for the second series). The description does not mention the color of bracts and sepals. Flowers, leaves and bracts seem to be larger than in *P.crenata* and *P.emiliae*, but comparable with the observations of Tillett (2003) for *P.riparia*.

As compared to all species descriptions mentioned above, that of *P.ambigua* is only differentiated by the color of the bracts, petals and sepals.

### Comparative analysis of herbarium and live specimens

Table 2 presents a comparison of the 43 herbarium specimens cited in the descriptions of *P.riparia* and its presumed synonyms, based on diagnostic traits. The observations of live specimens from two cited populations of *P.crenata* (Rome specimens) are also presented. Twenty-three herbarium specimens that do not match the syntype *Spruce 2191*, or that cannot be compared to it, are highlighted in bold font. Fourteen of them were assigned to other species of series *Laurifoliae*.

**Table 2.** Comparison of herbarium materials referenced in the descriptions of *P.riparia*, *P.emiliae*, *P.crenata*, *P.fernandezii*, *P.pergrandis* and *P.ambigua*. Two specimens of *P.crenata* from French Guiana have been replaced by field observations on populations at the collecting site. Question marks indicate that the trait could not be determined with confidence (e.g. pubescence on voucher scans). Dashes indicate that it could not be observed on the voucher (e.g. floral traits on sterile vouchers).

Specimen	Origin	Authors' determination	Petiolar gland position	Pseudo-raceme	Pubescence	Bracts size	Bract color	Perianth color	Outer series of filaments	Observations
Master (1872): *P.riparia*										
Spruce2191 (ST)	Brazil	* P. riparia *	middle	yes	peduncle - bracts - hypanthium - exterior of sepals	3 × 2 cm	reddish	white	Unequal, the outermost filiform and short, next one thicker and longer	
Martius 3228 (LT, IT)	Brazil	* P. riparia *	below middle	yes	bracts - ovary - fruit	3 × 2 cm	reddish pink	pink inside, white outside	"the outermost filiform, the second one thicker" (Latin handnote from Martius)	
Killip (1938): *P.riparia*										
Ducke 17338	Brazil, Para	* P. riparia *	middle	yes	?	4 × 2 cm	reddish	white	One, the outermost being completely atrophied	corona banded white and violet, androgynophore white
**Ducke 24044**	**Brazil, Amazonas**	** P. cf. laurifolia **	**apex**	**no**	?	–	**green**	**violet**	**Two unequal**	**no inner filaments, fruit acuminate**
**Killip 26307**	**Peru**	**impossible**	**middle**	–	–	–	–	–	–	**sterile plant, *P.riparia* or *P.ambigua***?
Killip 26683	Peru	* P. riparia *	below middle	–	–	–	–	–	–	"flowers blue and white" (label)
**Killip 28214**	**Peru**	**impossible**	**near apex**	–	–	–	–	–	–	**sterile plant**
**Killip 28940**	**Peru**	**impossible**	**middle**	–	–	–	–	–	–	**sterile plant, *P.riparia* or *P.ambigua***?
**Killip 29012**	**Peru**	**impossible**	**middle**	–	–	**4 × 2 cm**	–	–	–	**fruit orange, *P.riparia* or *P.ambigua***?
Klug 3897	Peru	* P. riparia *	middle	yes	peduncle - bracts - calyx - petioles of pseudoraceme	2 × 1.5 cm	reddish	white	one, the outermost being completely atrophied	
Klug 4037	Peru	* P. riparia *	middle	yes	?	–	–	grayish	one, the outermost being completely atrophied	
**Spruce 1172**	**Brazil**	** P. cf. laurifolia **	**apex**	**no**	**peduncle**	–	–	–	–	
**Spruce 1394**	**Brazil**	*** P. laurifolia ***	**apex**	**no**	?	–	**green**	**red**	**two unequal**	**no inner filaments**
**Spruce 3390**	–	*** P. phellos ***	**apex**	**no**	?	**1.5 × 0.5 cm**	**green**	**red**	**two unequal**	**corky stems**
**Swallen 3390**	**Brazil, Para**	*** P. laurifolia ***	**apex**	**no**	?	–	**green**	**purple**	**two unequal**	
**Williams 1392**	**Peru**	**impossible**	**below middle**	–	–	–	–	–	–	**sterile plant, *P.riparia* or *P.ambigua***?
Williams 1440	Peru	* P. riparia *	middle	yes	–	–	reddish	white	one, the outermost being completely atrophied	
**Williams 3126**	**Peru**	**impossible**	**middle**	**no**	**fruits - peduncles**	–	–	–	–	***P.riparia* or *P.ambigua***?
**Williams 5637**	**Peru**	*** P. venusta ***	**apex**	**no**	?	**4 × 2.2 cm**	**green**	**purple red**	–	**triangular sepals and petals, coriaceous leaves**
**Williams 5848**	**Peru**	** P. cf. venusta **	**below apex**	**no**	–	–	**green**	–	–	**coriaceous leaves**
**Williams 6300**	**Peru**	**impossible**	**below apex**	–	–	–	–	–	–	**sterile plant, *P.riparia* or *P.ambigua***?
**Williams 6378**	**Peru**	*** P. venusta ***	**near apex**	**no**	**ovary**	–	**green**	–	**two unequal series**	
Williams 7876	Peru	* P. riparia *	middle	no	ovary - peduncle - bracts	3 × 1.5 cm	reddish	white	Unequal, the outermost filiform and short, next one thicker and longer	
Williams 7996	Peru	* P. riparia *	below middle	yes	peduncle - bracts - stipule and petioles of pseudoraceme	3.5 × 2 cm	reddish	–	–	
Sacco (1966): *P.emiliae*										
Kuhlmann 1066 (HT)	Brazil, Mato Grosso	* P. riparia *	below middle	yes	ovary - peduncle - bracts- hypanthium - petioles of pseudoraceme	2.5–3.5 × 1.5–2.5 cm	white	white	Unequal, the outermost filiform and short, next one thicker and longer	short inner series (1–2 mm) in the hypanthium
Kuhlmann 1064 (PT)	Brazil, Mato Grosso	* P. riparia *	below middle	yes	ovary - peduncles - bracts-hypanthium - petiole of pseudoraceme	2.5–3.5 × 1.5–2.5 cm	white	white	Unequal, the outermost filiform and short, next one thicker and longer	short inner series (1–2 mm) in the hypanthium
Feuillet and Cremers (1984): P.crenata - vouchers										
Feuillet 573	French Guiana	* P. riparia *	middle	yes	peduncles - calyx - bracts - petioles of pseudoraceme	4–5 × 2–3 cm	white	white	Unequal, the outermost filiform and short, next one thicker and longer	
Prevost 563	French Guiana	* P. riparia *	below middle	yes	peduncle - bracts - fruit	5–6 × 4 cm	–	–	–	fruit green with white spots
Feuillet and Cremers (1984): P.crenata - live specimens from originally collected populations										
Rome specimens	French Guiana	* P. riparia *	below middle	yes	peduncles - calyx - bracts - petioles of pseudoraceme	4.5–6.2 × 2.7–4.3 cm	white to pink	white to greenish white	Unequal, the outermost filiform and short or absent, the second one thicker and longer	intermediate series atrophied and the innermost slightly longer; operculum membranous, horizontal, with margin recurved, crenulate
Holm-Nielsen et al. (1988): *P.riparia*										
**Holm-Nielsen 1040**	**Ecuador**	*** P. tolimana ***	**below apex**	**no**	?	**1.5 × 0.5 cm**	**reddish**	**lilac**	**two subequal series**	**bracts short and acute**
**Knapp 6242**	**Ecuador**	*** P. tolimana ***	**apex**	**no**	?	**1.5 × 0.4 cm**	**reddish**	**pinkish lavender**	**two subequal series**	**bracts short and acute, inner series oriented towards the androgynophore, covering the entrance to the hypanthium**
Cervi (1997): *P.riparia*										
**Schultes 9900**	**Brazil, Amazonas**	*** P. phellos ***	**apex**	–	?	**3 × 1 cm**	**green**	–	–	**corky stems**
**Spruce 1394**	**Brazil, Amazonas**	*** P. laurifolia ***	**see above, among materials cited by Killip (1938)**							
**Spruce 1172**	**Brazil, Amazonas**	** P. cf. laurifolia **								
**Spruce 3390**		*** P. phellos ***								
Ducke 17338	Brazil, Para	* P. riparia *	see above, among materials cited by Holm-Nielsen et al. (1988)							
**Allen 3340**	**Colombia**	*** P. laurifolia ***	**apex**	**no**	?	**3 × 1.5 cm**	**green**	**lavender**	**two unequal series**	–
**Jativa 439**	**Ecuador**	*** P. ambigua ***	**middle**	**yes**	**ovary**	**4.5 × 2.5 cm**	**green**	**petals white outside, pink inside**	–	
**Warush BBAE86**	**Ecuador**	**impossible**	**below middle**	**no**	**fruits - peduncles**	–	–	–	–	***P.riparia* or *P.ambigua***?
**Holm-Nielsen 1040**	**Ecuador**	*** P. tolimana ***	**see above, among materials cited by Holm-Nielsen et al. (1988)**							
**Smith 3157**	**Guyana**	*** P. laurifolia ***	**apex**		**bracts - peduncles**	**3.5 × 2 cm**	**green**	**red**	**two unequal series**	
**Ancuash 506**	**Peru**	**impossible**	**middle**	**yes**	**fruits - peduncles**	–	–	–	–	**fruit with a uniform color, could be *P.ambigua***
Schunke 907	Peru	* P. riparia *	below middle	yes	?	2.5 × 1.5 cm	reddish	–	one, the outermost being completely atrophied	
Revilla 241	Peru	* P. riparia *	below middle	yes	peduncles - bracts - calyx - fruit	2.5 × 1.5 cm	reddish	white	Unequal, the outermost filiform and short, next one thicker and longer	long peduncles
Klug 3897	Peru	* P. riparia *	see above, among materials cited by Killip (1938)							
Schunke 3555	Peru	* P. riparia *	middle	yes	?	1.5 × 1 cm	purple	–	Unequal, the outermost filiform and short, next one thicker and longer	fruit pale green, with white spots
Schunke 3579	Peru	* P. riparia *	middle	yes	?	2.5 × 1.5 cm	reddish purple	–	one, the outermost being completely atrophied	immature fruits green with white dots
Schunke 2112	Peru	* P. riparia *	middle	yes	?	3 × 1.5 cm	violet	–	one, the outermost being completely atrophied	
Holm-Nielsen and Lawesson (1987): *P.pergrandis*										
Harling 13771	Ecuador	* P. riparia *	middle	yes	?	3.5–4.5 × 3–4 cm	violet	more or less white	one, the outermost being completely atrophied	inner series of filaments reduced, filaments cross-striped in white and dark violet
Escobar (1989): P.fernandezii										
Fernández Casas 3341	Bolivia	P. riparia	middle	yes	peduncles - bracts - calyx - petioles of pseudoraceme (some trichomes on leaves of pseudoracemes)	2.5 × 1.5 cm	white	white	Unequal, the outermost filiform and short, next one thicker and longer	

The two collections used in the original description of *P.riparia* provide complementary information. *Spruce 2191* is the most complete; on the sample conserved in Paris, the corona of its flowers has a single series of well-developed filaments (about 35 mm long), whereas the outermost series is severely reduced (slender filaments ca. 5–10 mm). Its bracts and the abaxial sepal surface are slightly pubescent. The bracts are reddish (confirmed by a handwritten note on the Kew specimen). Two samples belong to *Martius 3228*. The first one has very degraded flowers and it is impossible to see the series of external filaments and the internal structure. However, a handwritten note by Martius mentions the presence of two series of main filaments, the outermost being filiform and the second one thicker; the corolla is described as pink outside and white inside, and the bracts as pink to red, which is consistent with the reddish color of their dry remains. The second sample bears long peduncles, however the flowers themselves are lacking. In these samples, the bracts, hypanthium, peduncles, and the abaxial face of the sepals are pubescent.

Out of the 22 specimens mentioned by Killip (1938) in his description of *P.riparia*, six cannot be identified (including four of the five sterile specimens he collected himself) and seven differ from the original description and the syntypes by the position of petiolar glands, the color of perianth and bracts, the presence of cork on the stem, showing that they belong to other species of series *Laurifoliae*. Finally, only seven specimens could be maintained unambiguously in *P.riparia*. Whenever observable (five specimens), the outermost series of filaments is shorter than the second series. In *Williams 7876*, the outermost series is longer than in *Spruce 2191*, but still much reduced as compared to the second series. In the four other cases (*Williams 1440*, *Klug 4037*, *Klug 3897* and *Ducke 17338*), it is even absent. Three specimens present pubescence on floral parts. *Klug 3897* and *Williams 7996* also exhibit pubescence on the petioles of pseudoracemes. *Williams 7876* shows no pseudoraceme.

The description of *P.emiliae* is based on only two herbarium specimens (*Kuhlmann 1066* and *Kuhlmann 1064*) collected from the same locality (margins of Rio Arinos, Mato Grosso). They are very similar to Spruce’s syntype of *P.riparia*, with the exception of bract color (white vs. reddish in the syntype). As indicated in Sacco’s description, the outermost corona filaments are shorter than in the second series. As in three specimens cited in Killip’s description, the ovary, peduncles, bracts and petioles of pseudoracemes are pubescent.

**Figure 1.**
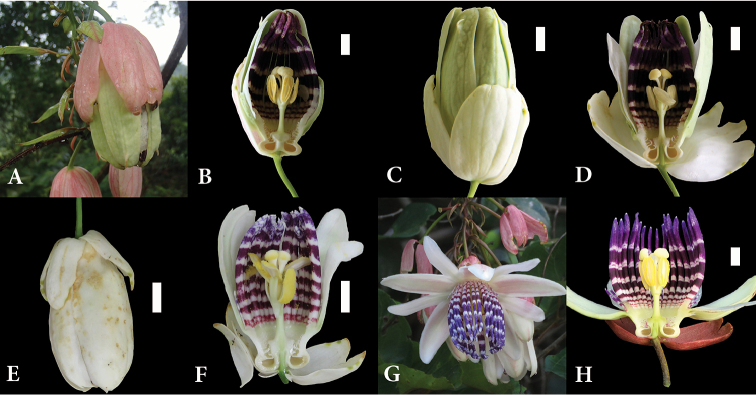
Comparison between flowers of *P.riparia* and *P.crenata.***A–C** flowers of *P.riparia* from Alta Floresta, Mato Grosso, Brazil (photo: Rich Hoyer) **D** flower of *P.riparia* from Marituba, Para, Brazil (photo: Luis Otavio Adão Teixeiro) **E–H** flowers of *P.crenata* from Cacao village, French Guiana. Flower size is indicated by 1 cm white bars.

In the description of *P.crenata*, Feuillet and Cremers (1984) refer to four herbarium specimens. The holotype (*Feuillet 573*) is similar to specimens of *P.emiliae* with white bracts again. It shows flowers gathered in pseudoracemes; pubescence on the peduncle, calyx, bracts, pseudoraceme petioles; and, contrary to the description of Feuillet and Cremers, two unequal series of filaments, with a shorter outermost series. *Prevost 563* only shows leaves with two glands below the petiole middle and a green fruit with white dots, while *Cremers 4294* and *Grenand 1825* were not available for examination. However, the corresponding localities mentioned in the publication, near the Cacao village and on the Regina road, were prospected. There, we observed several specimens with pink bracts in populations where the white bract phenotype dominates. This situation compares with that observed by Rich Hoyer, whose photographs document such polymorphism in Brazilian populations of *P.riparia* (Figure 1). Our field observations in French Guiana also confirm that *P.crenata* produces pseudo-racemes of flowers with large and pubescent sepals and show that its corona has two series of external filaments of different length, with an outer series that can even be absent (Figure 1 and Table 2). Field measures display a wide variability for quantitative traits in general.

Both specimens mentioned by Holm-Nielsen et al. (1988) in their description of *P.riparia*, *Holm-Nielsen 1040* and *Knapp 6242*, correspond to *P.tolimana*. This species can be easily distinguished from other Andean species of series *Laurifoliae* by its petiole with two glands at apex (or just below), very short and acute bracts, light pink to lilac perianth, two subequal series of corona with very slender filaments, and the innermost series that closes the entrance of the nectary chamber.

Out of the 20 herbarium specimens cited in the description of *P.riparia* by Cervi (1997), nine are misidentified and correspond to other species of series *Laurifoliae* (*Schultes 9900*, *Spruce 1394*, *Spruce 1172*, *Spruce 1394* and *Spruce 3390*, *Allen 3340*, *Jativa 439*, *Holm-Nielsen 1040* and *Smith 3157*), two cannot be identified (*Warush BBAE86*, *Ancuash 506*), as they are reduced to a stem with a few leaves and collected in areas where several closely related species coexist. *Pires 14246*, *Mathias 3982* and *Archer 3309* could not be verified. Contrary to Cervi’s description of *P.riparia*, *Ducke 17338*, *Schunke 907*, *Schunke 3576*, *Schunke 2112*, and *Klug 3897* exhibit only one outer series of filaments, the outermost one being completely absent, while *Revilla 241* and *Schunke 3555* show two unequal outer series of filaments (the outermost filiform and short, the second thicker and longer).

On *Harling 13771*, the holotype of *P.pergrandis*, the leaves bear two glands below the middle of petiole. Flowers, surrounded by three violet bracts, are gathered in pseudoracemes. They have a white perianth with one outer series of long filaments (the outermost filaments being vestigial), cross-striped in white and dark violet. The inner series are reduced (2–3 mm long). The pubescence of the plant could not be observed. Thus, *Harling 13771* belongs to *P.riparia*, as assessed from all the traits presented above: submedian petiolar glands, the occurrence of pseudo-racemes, colored bracts, white perianth, and a reduced outermost series of corona filaments.

Wide variation was observed for bract dimensions within the sets of specimens used by the different authors for the different taxa (see also bract size variation within populations in Figure 1), so this variation could not be related to any particular taxon.

### Analysis of distribution

A total of 83 specimens and live populations were georeferenced, 62 for *P.riparia* and its presumed synonyms, and 20 for *P.ambigua* (Table 3). In 59 cases of the former group, label information or precise location allowed inferences on the habitat corresponding to the collection. For their large majority, this habitat appears clearly humid (in the vicinity of flooded areas or water courses; 46 cases), and even frankly riparian in 32 cases (52%). The proportion of riparian collection sites was very similar for *P.riparia* and its four presumed synonyms. In comparison, 22% of examined *P.ambigua* specimens occupy a riparian habitat.

**Table 3.** Distribution of the georeferenced sample used for Ecological Niche Modeling and habitat information retrieved from voucher labels and aerial photographs (when geographic coordinates are very precise).

Species	Specimens/obs.	Ecological info.	Humid habitat	Riparian habitat
* P. riparia *	38	35	28	19
* P. emiliae *	3	3	2	2
* P. crenata *	12	12	8	5
* P. fernandezii *	2	2	2	1
* P. pergrandis *	7	7	6	5
Total	62	59	46	32
* P. ambigua *	20	18	5	4

**Figure 2.**
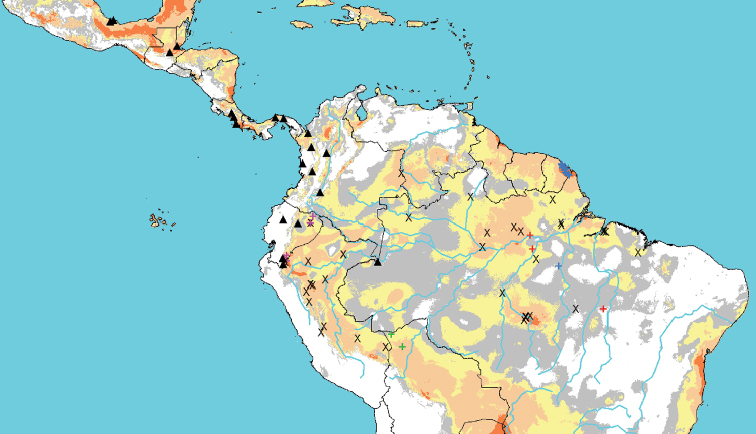
Distribution of examined specimens of *P.riparia* (X), *P.emiliae* (red cross), *P crenata* (blue cross), *P.fernandezii* (green cross), *P.pergrandis* (purple cross), and the bioclimatic distribution model, highlighting climates that are marginal (grey), favorable (light yellow), very favorable (light orange) or excellent (bright orange). Black triangles represent distribution of examined specimens of *P.ambigua*.

Figure 2 presents the geographic distribution of these collections and the bioclimatic distribution model obtained with the MAXENT software. The predicted range of *P.riparia* and its presumed synonyms includes the Guianas and the basins of the Amazon and southern Orinoco. The examined specimens broadly cover most of this range, except for the western Guianas (Suriname and Guyana) and most of northeastern Bolivia. *Passiflorariparia* has not been reported in regions covered by other great drainage systems, as the Magdalena/Cauca rivers in Colombia and the Paraguay/Paraná rivers in southern Brazil, although the latter seems to offer considerable extensions of favorable habitats. In contrast, *P.ambigua* is mostly present in the smaller drainage systems of the South American and Central American Andes. Only one of the specimens examined for this species was collected in the Amazon basin, near the triple border between Brazil, Peru and Colombia. Thus, the two taxa appear marginally sympatric.

**Figure 3.**
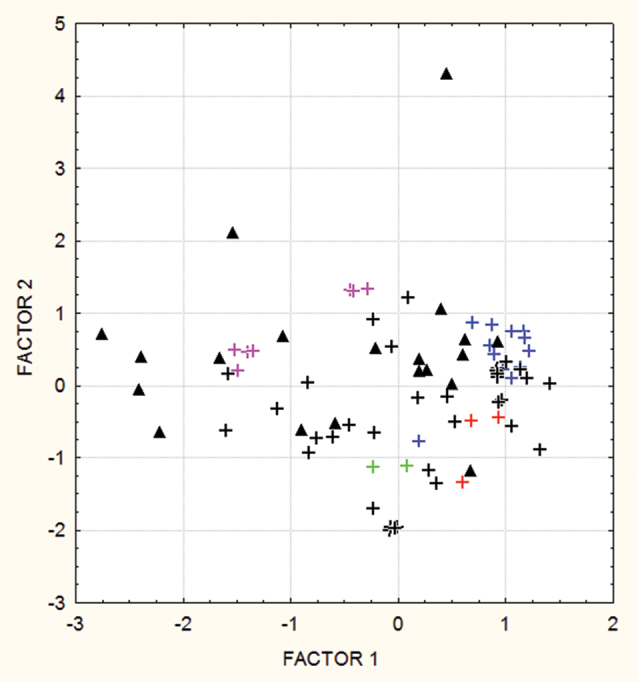
Representation of the climatic envelopes of *P.riparia* and its presumed synonyms in the principal plane of the PCA (same color code as for Figure 2). *Passifloraambigua* collections are represented by black triangles.

Table 4 and Figure 3 present the result of the PCA on the bioclimatic factors that contribute to the model with useful information that is not present in the other variables, according to the MAXENT jackknife test. Figure 3 presents the dispersion of the examined specimens in the principal plane, where factor 1 is more positively correlated with temperatures and factor 2 with precipitations (Table 4). The parallelism between the climatic and geographic spaces suggests that the climatic parameters that affect *P.riparia* and its presumed synonyms vary rather regularly across Amazonia. Thus, at first sight, *P.crenata* and *P.pergrandis* may appear relatively marginal, in both the geographical and climatic distributions. However, there is a clear continuity in the environmental space, and, compared to *P.riparia* specimens, these two taxa present no exceptional values in the bioclimatic principal plane. The bioclimatic envelope of *P.ambigua* appears much wider than that of *P.riparia* and its presumed synonyms. On one hand, it encompasses most of the *P.riparia* envelope, presenting very similar characteristics for precipitation, except for an outlier that corresponds to a specimen collected in the extreme conditions of the Colombian Chocó, under ca. 7 m annual rainfall. On the other hand it extends to habitats with cooler temperatures (to the left of the principal plane), which correspond to collecting sites at elevations above 1000 m in the Andes.

**Table 4.** Principal component analysis on the bioclimatic variables contributing to the MAXENT distribution model of *P.riparia*, *P.emiliae*, *P.crenata*, *P.fernandezii*, and *P.pergrandis*. Factor loadings.

Bioclimatic variable	Factor 1	Factor 2	Factor 3
2-Mean diurnal range	-0.45	-0.68	-0.17
5-Max. temp. of warmest month	**0.71**	-0.48	-0.28
6-Min. temp. of coldest month	**0.85**	0.33	-0.33
8-Mean temp. of wettest quarter	**0.86**	-0.12	-0.36
13-Precipitation of wettest month	0.55	0.40	0.63
14-Precipitation of driest month	-0.26	**0.85**	-0.33
15-Precipitation seasonality	0.51	-0.56	0.61
18-Precipitation of warmest quarter	-0.35	0.57	0.21
19-Precipitation of coldest quarter	0.52	0.67	0.09
Explained variance	3.19	2.80	1.28
Proportion of total	0.35	0.31	0.14

## Discussion

Most of the confusion in the definition of *P.riparia* has arisen from the incomplete description by Masters (1872), in the very difficult context of the highly uniform series *Laurifoliae*, and from the mention of two equal series of outer corona filaments by Killip (1938), which was reproduced in the following descriptions of Cervi (1997) and Tillett (2003) (although the latter mentioned the occasional loss of the outermost series). This ‘two equal outer series’ problem originated from the ambiguous iconography accompanying the description of Masters (1872), which is inconsistent with the syntype *Spruce 2191*, and all other interpretable specimens mentioned in the literature. Indeed, our study of the specimens examined by Killip (1938) and Cervi (1997) shows that, whenever the relative length of outer corona filaments is observable, i.e. in 12 of them, their outer series is reduced, or even absent in nine of them. This clearly indicates that Killip (1938) and Cervi (1997) had not observed themselves the combination of two equal series of outer filaments with multiple series of very short inner filaments and other typical traits as white to red bracts and white corollas.

In 1966, Sacco realized the inconsistency between Killip’s description of *P.riparia* and two Kuhlmann specimens of this species where he could observe the reduction of the outermost series of filaments. But he used them as types for the description of a new species, *P.emiliae*, logically differentiated from *P.riparia* by the outer corona structure. It seems that this new species description remained confidential and only attracted the attention of Cervi, who reclassified a few other exemplars *from P.riparia* to *P.emiliae*, such as *Ducke 21311*, *Kuhlmann sn* (*R136313*). Later, in 1997, he changed his mind and followed Holm-Nielsen et al. (1988) in considering it a synonym of *P.ambigua*. Table 1 shows that the description of *P.emiliae* only differs from those of *P.riparia* by the pubescence of the flower; however, this trait is also found in many examined specimens of *P.riparia*, including the syntypes (Table 2), as well as in the other presumed synonyms, *P.crenata*, *P.fernandezii* and *P.pergrandis*.

Originally, *P.crenata* was described as an endemic of French Guiana, distinguished from *P.riparia* by its white bracts and the type of inflorescence. However, bract color at anthesis varies between white and deep red in populations from French Guiana and from Brazil (Figure 1, Table 2) and the type specimens of *P.emiliae* also present white bracts. In the examined specimens from Ecuador and Peru, bract color varied from red to purple and violet. The presence of single axillary flowers or of pseudoracemes is also a variable trait, even at the intra-individual level, as noted in the descriptions of *P.riparia* by Tillett (2003), *P.emiliae* by Sacco (1966), and observed by ourselves in *P.crenata*. Furthermore, other species of series *Laurifoliae* present both solitary flowers and pseudoracemes, as *P.ambigua*, *P.popenovii* Killip, *P.gabrielliana* Vanderpl., *P.capparidifolia* Killip, *P.killipiana* Cuatrec., *P.phellos* Feuillet, *P.laurifolia*. Thus, no traits allow distinguishing *P.crenata* from *P.riparia* and *P.emiliae*.

The two more recent descriptions of *P.pergrandis* and *P.fernandezii* did not present new variations in any of the characters discussed above. Finally, we can conclude that the differences in the descriptions of *P.riparia*, *P.emiliae*, *P.crenata*, *P.pergrandis* and *P.fernandezii* are either related to the confusion introduced by the iconography associated with the original description or with the imprecisions related to the initial observation of two dry specimens. More details and variation have logically been documented after the examination of specimens from a wider geographic range, as well as the direct observations on living materials. The variations observed among specimens of *P.riparia* encompass the variations between this taxon on one hand and *P.crenata*, *P.emiliae*, *P.fernandezii* or *P.pergrandis* on the other hand. Furthermore, most specimens from the latter taxa have been collected under very similar habitats, dominated by lowland tropical climates in riparian habitats (Table 3, Figure 3), further justifying their placement as synonyms of *P.riparia*. Only the description of *P.riparia* in the Flora of Ecuador is clearly different, as it includes the mention of a lilac corolla, two extremely long series of outer filaments and the presence of an inner series of 1 cm erect filaments, a trait combination indicating that Holm-Nielsen et al. (1988) described *P.tolimana* instead of *P.riparia*.

*Passifloraambigua* appears very similar to *P.riparia*, mostly differing in the color of bracts (green) and the corolla (lilac), but its distribution and ecology are different, with a much lesser frequency of riparian habitats and a capacity to thrive at much higher elevations, up to hillsides in the Andes of tropical South America and Central America.

Gathering *P.riparia*, *P.crenata*, *P.emiliae*, *P.fernandezii* and *P.pergrandis* into a single species imposes a new description, taking into account the reduction of the outermost series of corona filaments and variation observed in other traits, on our whole sample. In the following treatment, the lectotype and isotype chosen by Lawesson are logically retained, however, as its damaged flowers do not allow the observation of the corona structure, we choose as epitype the syntype *Spruce 2191*, i.e., the much better-preserved specimen that was observed by Masters for the original description.

### Taxonomic treatment

#### Passiflora riparia

Taxon classificationPlantaeMalpighialesPassifloraceae

Mart. ex Mast. in Martius, Fl. Bras. 13(1): 599. 1872

Passiflora emiliae Sacco, Boletim do Museu Nacional de Rio de Janeiro. Botanica 32: 1–5. 1966. Type: Brazil. Mato Grosso, Rio Arinos, Dec. 1914, Kuhlmann 1066 (holotype, R!), Kuhlmann 1064 (paratype, R!).
Passifloracrenata Feuillet & Cremers, Proceedings of the Koninklijke Nederlandse Akademie van Wetenschappen, Series C: Biological and Medical Sciences 87(4): 378. 1984. Type: French Guiana. Road between Roura and the Kaw mountain, 24 Jan. 1983, *Feuillet 573* (holotype, CAY!; isotype, BR, P!, U, US).
Passiflora pergrandis Holm-Niels. & Lawesson, Annals of the Missouri Botanical Garden 74(3): 501, f. 4. 1987. Type: Ecuador. Zamora-Chinchipe: Zamora –Gualaquiza road, 5 km N of Cumbaraza, 900 m, 20 Apr. 1974, *Harling 13771* (holotype, GB! ; isotype, AAU)
Passiflora fernandezii L. K. Escobar, Phytologia 66(1): 80–81. 1989. Type: Bolivia. Pando: Nicolas Suarez: between Porvenir and Cachuelita, along the trail, 19 Jan 1983, *F. Javier Fernández Casas 8341* (holotype, NY; isotypes, MO!, NY).

##### Type.

Brazil. Forest near mouth of Madeira River, Brazilian state of Amazonas (in silvis prope ostium fluvii “Madeira”), March 1819, *Martius 3228* (lectotype, M! designated by Holm-Nielsen et al. (1988); isotype, M!). São Gabriel da Cachoeira, Amazonas, April 1852, *Spruce 2191* (epitype, P!, isoepitypes M!, K!, designated here).

Woody liana. Stem terete to subangulate, glabrous to slightly pubescent (on young parts or pseudoracemes), and green; internodes 4–48 cm. Tendrils glabrous, green. Stipules setaceous to narrow linear, generally aristate, yellow green to brown purple, eglandular to glandular (1–2 nectaries), 8.8–18.1 × 0.2–1.4 mm (including an arista 0–2.7 mm), early deciduous. Petiole 1.3–3.8 cm long, green to dark green, slightly canaliculate adaxially, glabrous (pubescent on pseudoracemes), bearing two conspicuous oval sessile glands (about 1 mm long), at or below the middle (0.4–1.3 cm from petiole base). Leaves simple, unlobed, 10.5–21 × 5.5–11 cm, glabrous throughout, green to dark green, adaxial surface lustrous, cordate to rounded at base, obtuse to acute at apex, mucronate and acuminate; margin entire (rarely glandular-serrulate). Inflorescence axillary, sessile, 1-flowered. Peduncles terete, green, pubescent, 1.9–2.8 mm in diameter, 1.4–3.5 cm long; pedicel 6.5–10 mm long. Bracts deciduous (at fruit maturity), pubescent on both sides, white to dark purple or white and more or less pink-purple veined, concave, free to base, 2.5–6.2 × 1.4–4.3 cm, with 3–7 marginal sessile green nectaries in distal half. Flowers axillary, pendulous, 2.8–3.4 cm long (from the base of nectary chamber to the ovary apex), solitary or presented in clusters on pseudoracemes (short internode branches). Nectary chamber pubescent externally, white greenish outside and white inside, 14.8–20.3 mm in diameter, 4.5–11.9 mm in depth. Hypanthium campanulate, pubescent, white greenish outside and white inside, 15–20 mm long and 18–21 mm in diameter at the base of sepals. Sepals pubescent, oblate, 4.2–6.4 × 1.8–2.8 cm, adaxial surface white to slightly pink, abaxial surface white to white greenish, slightly keel-shaped in distal half with a short awn (3–5 mm long). Petals glabrous, oblate, 4.2–5.4 × 1.2–1.6 cm, white. Corona filaments in 6–9 series, banded white and purple to dark purple; two major outer series, slightly curved, unequal: outer series 0–18 mm, second series 43.9–55.4 mm; inner series 1–2 mm, curved filiform, white with purple tip, covering the interior of the hypanthium. Staminal filaments 8–11 mm long, white greenish. Ovary pubescent, white, 8–9 mm long; three styles, white, 9–12 mm long, stigmas white to cream. Androgynophore glabrous, greenish white, 14–17 mm long with an enlarged base about 10 mm wide. Operculum membranous, 4–5 mm long, recurved, shortly fimbriated at margin. Fruit obovoid, round in transversal section, pubescent, 3.6–7.3 cm long, about 2.5–4.8 cm in diameter; pericarp 0.5–1 cm thick; immature fruits green with fine white dots; mature fruits light orange, white spotted, with a sweet translucent pulp. Seeds obovoid, flat, with retuse apex, about 1 cm long.

## Conclusion

Following our morphological and ecological analyses, *P.emiliae*, *P.pergrandis*, *P.fernandezii* and *P.crenata* are placed as synonyms of *P.riparia*, which reduces the current number of species belonging to series *Laurifoliae* to 21. Like *Passifloranitida*, *P.laurifolia* or *P.ambigua*, *P.riparia* is a new example of a very widely distributed species in this series. In fact, its variability appears relatively limited in the context of its wide distribution.

The description of *P.riparia* in the Flora of Ecuador corresponds to *P.tolimana; Holm-Nielsen 1040* and *Knapp 6242* are the only known specimens from Ecuador for this species, hitherto considered endemic to Colombia. The determination of *Spruce 2191* under *P.ambigua* by Lawesson, endorsed by Holm-Nielsen et al. (1988), cannot be accepted, given that this specimen has white petals and sepals (vs. red to purple in *P.ambigua*).

## Supplementary Material

XML Treatment for Passiflora riparia

## Appendix

**Examined specimens**

***
P. ambigua
***

**BRAZIL**. **Amazonas**: Esperança, mouth of Javari River, non-flood forest, 4-2-1942, *Ducke 878* (MO). **COLOMBIA**. **Antioquia**: San Luis, 26 Jul. 1981, *Hoyos 132* (JAUM). Municipality of Frontino, Las Orquídeas National Park, 31 Jan. 1995, *Pipoly 18177* (JAUM). Municipality of Turbo, road of Tapón del Darién, 28 Feb. 1984, *Brand 947* (JAUM). **Chocó**: area of Baudó, 4 Feb. 1967, *Fuchs 21744* (COL). **Huila**: Municipality of Gigante, farm of Adonai Moreno, 20 Dec. 1976, *Escobar 435* (MO). **Meta**: Río Bravo village, La Cristalina stream, El Pital, 4 Mar. 1986, *Devia 1121* (MO). **COSTA RICA**. **Puntarenas**: Osa Península, 6 Jan. 1994, *Aguilar 2974* (CR). **Cartago**: Orosi, Navarro del Muñeco, 12 Apr. 1998, *Blanco 805* (USJ). **San José**: Vicinity of El General, Jan. 1939, *Skutch 3037* (US). **ECUADOR**. **Esmeraldas**: El Timbre, near Esmeraldas, 6 Aug. 1962, *Jativa 439* (US). **Napo**: Road Hollín-Loreto-Coca, Chaluayacu Community, 23 Dec. 1988, *Cerón 5772* (MO). **Pichincha**: Alluriquin, 19 Oct. 1921, *Werling 458* (QCA). **Zamora-Chinchipe**: Municipality of Zamora, Road Zamora-Loja, 29 Oct. 1991, *Palacios 8811* (MO). Palanda, Río Vergel Valley, 14 Nov. 2006, *Werff 22075* (MO). **GUATEMALA**. Secanquim, 14 May 1914, *Cook 79* (US). **HONDURAS.** Machaca, 8 Feb. 1934, *Schipp 1302* (F). **MEXICO**. **Veracruz**: Biological station of Los Tuxtlas, 7 Apr. 1971, *Calzada 230* (MEXU). Municipality of San Andrés Tuxtla, south of Ebitrolotu, 30 APr. 1973, *Villegas Herrera 112* (MEXU). **PANAMA. Colón**: Santa Rita Ridge, east of transisthmian highway, 16 Dec. 1972, *Gentry 6561* (MO). **Panamá**: just before la Eneida along new trail which begins exactly beside López House, 8 March 1968, *Correa 825* (PMA). El Llano-Carti Road, 18 Apr. 1981, *Sytsma 4027* (MO). **PERU**. **Loreto**: Maynas, near Iquitos, in 1996, *Grandez 7837* (MO).

***
P. laurifolia
***

**BRAZIL. Bahia**: Barra, *Spruce 1394* (P). **Pará**: Santarem, 19 Jan. 1934, *Swallen 3309* (US). **COLOMBIA. Vaupés**: Vicinity of Mitú, Rio Vaupés, 22 Feb. 1945, *Allen 3340* (US). **GUYANA**. Western extremity of Kanuku Mountains, in drainage of Takutu River, 4 March 1938, *Smith 3157* (U).

***
P. phellos
***

**BRAZIL**. **Amazonas**: Uanadona, near mouth of Rio Dimiti, 10 May 1948, *Schultes 9900* (K). **VENEZUELA**. Río Pacimini, Feb. 1854, *Spruce 3390* (K).

***
P. riparia
***

**BOLIVIA**. **La Paz**: Prov. Iturralde, Siete Cielos, Rio Manupare, 4 June 1987, *Solomon 16923* (MO). **BRAZIL. Acre**: Municipality of Plácido de Castro, Rio Abunã, 10 Jan. 1995, *Figueiredo 506* (NY). **Amapá**: National park of Tumucumaque, Rio Jari, 21 Feb. 2013, *Hopkins 2284* (INPA). Municipality of Monção, basin of the Rio Turiaçu, Ka’apor Indian Reserve, 12 Feb. 1985, *Balée 794* (MO), *Balée 810* (MO). Amazonas: Presidente Figueredo, Nazaré locality, Rio Uatumã between Rio Pitinga and Rio Uatumã, 18 March 1986, *Ferreira 6808* (NY). **Amazonas**: Mouth of Rio Madeira, *Martius 3228* (M). São Gabriel da Cachoeira near the Rio Negro, Apr. 1852, *Spruce 2191* (P). **Pará**: Ruropolis Presidente Medici, 9 Feb. 1976, *Bamps 5341* (K). Municipality of Almeirim, Mte. Dourado, road Mte Dourado-Munguba, 10 Feb. 1986, *Pires 784* (INPA), Mt. Dourado, 20 June 1988, *Pires 2227* (NY). Banks of Rio São Manoel, 14 Jan. 1952, *Pires 3931*. Belem, 29 Dec. 1922, *Ducke 17338* (US). Rio Xingu, Island of Piracui, 22 Oct. 1986, *Souza 449*. **Goias**: ca. 2 km S. of Guará, 19 March 1968, *Irwin 21451* (NY). **Mato Grosso**: Rio Arinos, Dec. 1914, *Kuhlmann 1066* (R), *Kuhlmann 1064* (R). Novo Mundo, Forest Reserve of Rio Cristalino, 11 Feb. 2008, *Zappi 1194*, margin of Rio Cristalino, 29 Nov. 1996, *Dubs 2335* (K). Alta floresta, private property of environmental preservation, 10 Nov. 2006, *Sasaki 1087* (K), 13 Dec. 2006, *Sasaki 1199* (INPA), 23 Jan. 2007, *Sasaki 1390* (INPA). **COLOMBIA. Guainía**: confluence of Ríos Guaviare, Atabapo and Inirida, 23 Aug. 1975, *García-Barriga 20929* (COL). **ECUADOR. Sucumbios**: Cuyabena faunistic reserve, Rio Cuyabeno from outlet of Laguna Grande and 5 km upstream, 2 Apr. 1989, *Balslev 84715* (QCA). **Orellana**: National park Yasuní, south of Río Napo, 31 Jan. 1998, *Burnham 1628* (QCA), Río Tivacuno, 0.5 km upstream of confluence with Río Tiputini, 24 March 1998, *Burnham 1690* (QCNE). National Park Yasuní, 16 Feb. 2002, *Pérez 392* (QCA). **Zamora-Chinchipe**: Road Zamora-Gualaquiza, ca 5 km north of Cumbaraza, 20 Apr. 1974, *Harling 13771* (GB). Nangaritza, Río Tzenganga, 6 June 2005, *Quizhpe 1201* (MO). Hill about 2 km downstream from Campamento Shaime along Río Nangaritza, 15 Feb. 1994, *Werff 13084* (QCNE). **FRENCH GUIANA.** Road to Montsinery, 7 March 2017, *Rome 567* (P). Road between Cayenne and Cacao, 25 Feb. 2017, *Rome 559* (P). Road to Régina, 17 May 2008, *Rome 118* (LYJB), *Rome 119* (LYJB), *Rome 120* (LYJB), *Rome 121* (LYJB), *Rome 122* (LYJB). Road between Régina and St Georges, 10 Apr. 2008, *Rome 45* (LYJB), 19 May 2008, *Rome 129* (LYJB), *Rome 136* (LYJB), *Rome 138* (LYJB), *Rome 142* (LYJB). Road of Kaw Mountain, 24 Nov. 2009, *Rome 201* (LYJB), *Rome 202* (LYJB), 29 Jan. 2013, *Rome 400* (LYJB). Road to Tonnegrande, 30 Jan. 2013, *Rome 405* (LYJB), *Rome 406* (LYJB), *Rome 407* (LYJB), *Rome 408* (LYJB), *Rome 409* (LYJB). Kotika mount, 21 Feb. 2005, *Granville (de) 16896* (CAY). Municipality of Saint-Georges, Oyapock basin, 15 Aug. 1997, *Berton 250* (CAY). Kamuyene Kamuyene, 12 Apr. 2005, *Bourdy 3128* (CAY). Road of Kaw mountain, 24 Jan. 1983, *Feuillet 573* (CAY), 1 Jan. 1986, *Feuillet 2975* (CAY), 25 Aug. 2009, *Feuillet 17078* (CAY). Path between Roura and the Gabrielle Creek, 21 Apr. 1979, *Prevost 563* (CAY). Road to Régina, near Camp Hervo, 14 March 2009, *Vanderplanck 1605* (CAY). **PERU. Huánuco**: Prov. Pachitea, region of Pucallpa, western part of the “Sira montains” and adjacent lowland, 31 July 1988, *Wallnöfer 12-31788* (MO). Along Río Pachitea, 12 July 1967, *Schunke 2112* (US). **Junín**: Chanchamayo prov., El Bocaz, 20 Nov. 1982, *Vargas 64* (USM). **Loreto**: in lower forest at Neshuya, 2 Oct. 1965, *Schunke 907* (US). **Madre de Dios**: National Park Manu, Río Manu, 1 Oct. 1986, *Foster 11539* (F), *Foster 11558* (F). Tambopata prov., Las Piedras, Cusco Amazónico, 7 Oct. 1991, *Timaná 2457* (MO). **San Martín**: Mariscal Cáceres, mouth of Río Tocache, 6 Nov. 1969, *Schunke 3579* (US). San Martín, Boca Toma del Shicayo, N of Tarapoto along Río Shilcayo, 27 May 1986, *Alcorn 10* (MO). Juan Jui, Alto Río Huallaga, Oct. 1934, *Klug 3897* (MO). Chazuta, Río Huallaga, March 1935, *Klug 4037* (K). Mariscal Cáceres, Canyon of Huaquisha, right margin of Río Huallaga, 30 June 1974, *Schunke 7083* (MO). **Amazonas**: between Uchpayaco and Rimachi, banks of Río Pastaza, 30 July 1979, *Díaz 1283* (MO). **Pasco**: Oxapampa, Puerto Bermúdez, 14 July 1929, *Killip 26683* (US). Oxapampa, ca. 1 km from division of Villa Rica-Pto. Bermúdez road and Villa Rica-Palcazu road, on Palcazu branch, 15 Aug. 1984, *Knapp 6633* (MO). **Loreto**: Maynas, Iquitos, July 1929, *Williams 1440* (US), March 1930, *Williams 7996* (US). Yurimaguas, lower Río Huallaga, March 1930, *Williams 7876* (US). Maynas, district of Iquitos, Caserío, 25 Feb. 1976, *Revilla 241* (MO), Río Nanay near Astoria, 15 March 1973, *Rimachi 126* (USM). Tarapoto, Nov. 1902, *Ule 6547* (K). **Ucayali**: Pucapanga, margin of Río Ucayali, 20 Aug. 1965, *Sagástegui 5739* (HUT). **SURINAME.** Sipaliwini, vicinity of airstrip along Ulemari river, 1 May 1998, *Evans 2971* (MO).

***P.* sp.** (impossible or doubtful identification)

**BRAZIL. Amazonas**: Lake of Aleixo, in riparian forest, 3 Apr. 1932, *Ducke 24044* (US). Bahia, Barra, March 1851, *Spruce 1172* (P). **ECUADOR**. **Morona Santiago**: Shuar Pampants center, 10 Sept. 1986, *Warush RBAE86* (QCNE). **PERU**. **Amazonas**: Río Cenepa, 2 June 1973, *Ancuash 506* (US). **Junín**: Junín, Puerto Yesup, 10 July 1929, *Killip 26307* (US). **Loreto**: Alto Amazonas, Yurimaguas, lower Río Huallaga, 22 Aug. 1929, *Killip 28214* (US). Near Iquitos, July 1929, *Williams 1392* (US). La Victoria on the Amazon River, Aug. 1929, *Williams 3126* (US). Santa Rosa, Lower Río Huallaga below Yurimaguas, 1 sept. 1929, *Killip 28940* (US). Wooded banks of lower Río Huallaga, 5 Sept. 1929, *Killip 29012* (US). **San Martín**: Alto Río Huallaga, Dec. 1929, *Williams 6300* (US). Tarapoto, Alto Río Huallaga, Dec. 1929, *Williams 5848* (F).

***
P. tolimana
***

**ECUADOR**. **Napo**: 2 km W of Archidonia, 27 June 1968, *Holm-Nielsen 1040* (AAU). **Morona-Santiago**: ca. 32.5 km S. of Gualaquiza on road to Zamora, 4 Feb. 1984, *Knapp 6242* (US).

***
P. venusta
***

**PERU. San Martín**: Lamas, Dec. 1929, *Williams 6378* (US). Tarapoto, Alto Río Huallaga, Dec. 1929, *Williams 5637* (F).

**Index to Numbered Collections**

Aguilar, R. 2974 (ambigua)

Alcorn, P. 10 (riparia)

Allen, P. H. 3340 (laurifolia)

Ancuash, E. 506 (sp.)

Balée, W. L. 794, 810 (riparia)

Balslev, H. 84715 (riparia)

Bamps, P. 5341 (riparia)

Berton, M. E. 250 (riparia)

Blanco, M. 805 (ambigua)

Bourdy, G. 3128 (riparia)

Brand, J. 947 (ambigua)

Burnham, R. J. 1628, 1690 (riparia)

Calzada, J. I. 230 (ambigua)

Cerón, C. E. 5772 (ambigua)

Cook, O. F. 79 (ambigua)

Correa, M. D. 825 (ambigua)

Devia, W. 1121 (ambigua)

Díaz, C. 1283 (riparia)

Ducke, A. 17338 (P.riparia), 24044 ( sp.), 878 (ambigua)

Dubs, B. 2335 (riparia)

Escobar, L. 435 (ambigua)

Evans, R. 2971 (riparia)

Feuillet, C. 573, 2975, 17078 (riparia)

Ferreira, C. A. C. 6808 (riparia)

Figueiredo, C. 506 (riparia)

Foster, R. B. 11539 (riparia)

Fuchs, H. P. 21744 (ambigua)

García-Barriga, H. 20929 (riparia)

Gentry, A. 6561 (ambigua)

Grandez, C. 7837 (ambigua)

Granville (de), J. J. 16896 (riparia)

Harling, G. 13771 (riparia)

Holm-Nielsen, L. 1040 (tolimana)

Hopkins, M. J. G. 2284 (riparia)

Hoyos, S 132 (ambigua)

Irwin, H. S. 21451 (riparia)

Jativa, C. D. 439 (ambigua)

Killip, E. P. 24044, 26307, 28214, 28940, 29012 ( sp.), 26683 (riparia)

Klug, G. 3897, 4037 (riparia)

Knapp, S. 6633 (P.riparia), 6242 (tolimana)

Kuhlmann, J. G. 1066, 1064 (riparia)

Martius, C. F. P. 3228 (riparia)

Palacios, W. 8811 (ambigua)

Pérez, A. J. 392 (riparia)

Pipoly, J. 18177 (ambigua)

Pires, M. J. P. 784, 2227, 3931 (riparia)

Prevost, M. F. 563 (riparia)

Quizhpe, W. 1201 (riparia)

Revilla, J. 241 (riparia)

Rimachi, M. 126 (riparia)

Rome, M. 567, 559, 118, 119, 120, 121, 122, 45, 129, 136, 138, 142, 405, 406, 407, 408, 409 (riparia)

Sagástegui, A. 5739 (riparia)

Schunke, J. V. 907, 2112, 3555, 3579, 7083 (riparia)

Sasaki, D. 1087, 1199, 1390 (riparia)

Schipp, W. A. 1302 (ambigua)

Schultes, R. E. 9900 (phellos)

Skutch, A. F. 4037 (ambigua)

Smith, A. C. 3157 (laurifolia)

Solomon, J. C. 16923 (riparia)

Souza (de), S. A. M. 449 (riparia)

Spruce, R. 1172 (sp.), 1394 (laurifolia), 3390 (phellos), 2191 (riparia)

Swallen, J. R. 3309 (laurifolia)

Sytsma, K. J. 4027 (ambigua)

Timaná, M. 2457 (riparia)

Ule, E. 6547 (riparia)

Vanderplanck, R. J. R. 1605 (riparia)

Vargas, M. A. 64 (riparia)

Villegas Herrera, A. 112 (ambigua)

Wallnöfer, B. 12-31788 (riparia)

Warush, A. 86 (sp.)

Werff (van der), H. 13084 (riparia), 22075 (ambigua)

Werling, L. 458 (ambigua)

Williams, L. 1392, 3126, 5848, 6300 (sp.), 5637, 6378 (venusta), 1440, 7876, 7996 (riparia)

Zappi, D. 1194 (riparia)
